# The DmtA methyltransferase contributes to *Aspergillus flavus* conidiation, sclerotial production, aflatoxin biosynthesis and virulence

**DOI:** 10.1038/srep23259

**Published:** 2016-03-16

**Authors:** Kunlong Yang, Linlin Liang, Fanlei Ran, Yinghang Liu, Zhenguo Li, Huahui Lan, Peili Gao, Zhenhong Zhuang, Feng Zhang, Xinyi Nie, Shimuye Kalayu Yirga, Shihua Wang

**Affiliations:** 1 Key Laboratory of Pathogenic Fungi and Mycotoxins of Fujian Province, Key Laboratory of Biopesticide and Chemical Biology of Education Ministry, and School of Life Sciences, Fujian Agriculture and Forestry University, Fuzhou 350002, China

## Abstract

DNA methylation is essential for epigenetic regulation of gene transcription and development in many animals, plants and fungi. We investigated whether DNA methylation plays a role in the development and secondary metabolism of *Aspergillus flavus*, identified the DmtA methyltransferase from *A. flavus*, and produced a *dmtA* knock-out mutant by replacing the *dmtA* coding sequence with the *pyrG* selectable marker. The *A. flavus dmtA* null mutant lines produced white fluffy mycelium in liquid medium, and displayed a slightly flavescent conidial pigmentation compared with the normal yellow of the wild-type strain when grown on agar. The Δ*dmtA* lines exhibited decreased conidiation and aflatoxin (AF) biosynthesis, compared with the wild-type line, suggesting that the DmtA knock-out affected the transcriptional level of genes in the AF cluster. In particular, sclerotia development and host colonization were altered in the *dmtA* null mutants. Green fluorescent protein tagging at the C-terminus of DmtA showed that DmtA localized to the nucleus and cytoplasm. DNA methylation content measurements in the *dmtA* mutants revealed no widespread DNA methylation in the mutants or wild-type lines. Thus, our findings suggest that DmtA, apart from being a C-5 cytosine methyltransferase in *A. flavus*, contributes to asexual development, aflatoxin biosynthesis, sclerotial production and virulence.

DNA methylation at selected cytosine is an important epigenetic process that occurs in most prokaryotes and eukaryotes^1^,^2^. DNA methylation has been shown to play a vital role in many biological processes, including gene transcription regulation, transposable element silencing, genome imprinting, X-chromosome inactivation and development^1^,^3^. Abnormal DNA methylation is implicated in human diseases and development defects in plants^4^,^5^. Evidence that DNA methylation is associated with developmental processes in most higher eukaryotes is provided by the involvement of the maintenance DNMT1 DNA methyltransferase and the *de novo* DNMT3 DNA methyltransferase in development^1^,^6^,^7^.

Although the function of DNA methylation has been well-studied, much remains to be learned about DNA methyltransferases (DMTs). In eukaryotes, DMTs can be divided into five clans based on their structures and functions: the maintenance methyltransferase clan (DNMT1), the *de novo* methyltransferase clan (DNMT3), the plant-specific chromomethylase clan, the DNMT2 clan with weak DMT catalytic activity, and the fungal-specific DMT-like clan^8^,^9^. In fungi, numerous studies have sought to identify methylated genomic sites and their associated DMTs^1^,^10^,^11^,^12^. Two putative DMTs, Masc1 in *Ascobolus immerses* and RID (*RIP D*efective) in *Neurospora*, were shown to function in repeat-induced point (RIP) mutation, a process regarded as a genome defence system, and methylation induced mutation (MIP), respectively^8^,^13^. In *Aspergillus nidulans*, a DMT homologue called DmtA, which is essential for sexual development, has been identified^8^. However, DNA methylation in *Aspergillus* and especially in *A. flavus* is extremely low^11^. Therefore, the role and significance of these putative DMTs in DNA methylation remain unclear in *Aspergillus*.

The *Aspergillus* genus is a large, worldwide family of fungi with more than 185 known species, among which *A. flavus* is the most economically important because it contaminates seed crops and food-stuffs with the toxic and carcinogenic secondary metabolite, aflatoxin (AF)^14^,^15^,^16^. Ingestion of such contaminated food poses a significant threat to human and animal health because of its hepatotoxicity and immunotoxicity^15^,^17^,^18^. Given these detrimental properties, effective strategies are required to control AF synthesis in preharvest and postharvest seed crops. To date, despite much progress having been made in elucidating the mechanism of AF biosynthesis, the contribution of epigenetic modifications such as DNA methylation or the involvement of DMTs in AF metabolism are yet to be thoroughly studied^11^,^15^,^19^. In *A. flavus*, although the existence of DNA methylation is controversial, our former study has shown that use of the DMT inhibitor 5-azacytidine resulted in decreased AF production and concurrent morphological changes^15^, suggesting that DNA methylation or DMTs possibly play roles in AF metabolism and *A. flavus* development. This is surprising, because neither widespread DNA methylation nor active DMTs have been identified in *A. flavus*^11^. Here, we identified and cloned the putative DMT coding gene, *dmtA*. Accordingly, in order to investigate its potential function(s), we used gene knock-out to delete this gene from the *A. flavus* genome. Inactivation of *dmtA* inhibited AF biosynthesis and conidiation, and resulted in a change of seed infection.

## Results

### Identification and analysis of a C-5 cytosine methyltransferase in *Aspergillus*

**Figure 1:**
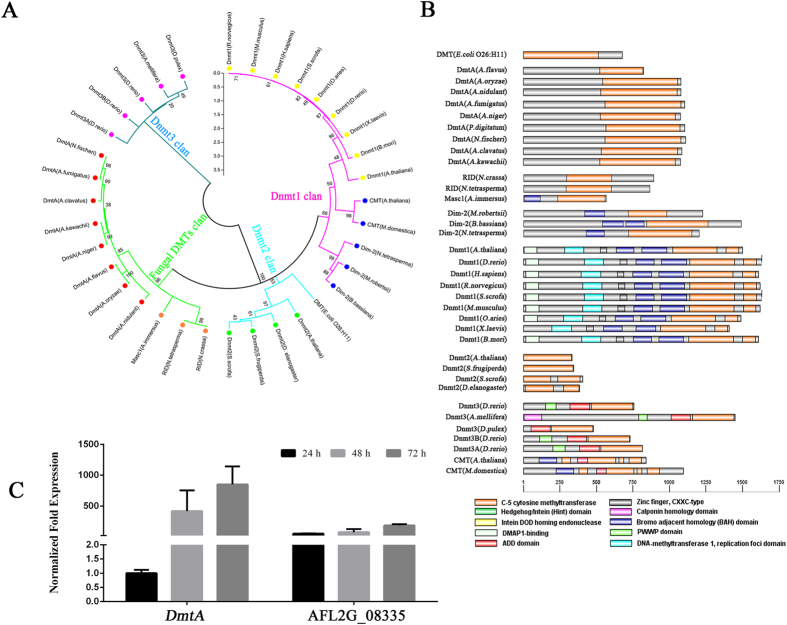
Phylogenetic and structure analysis of DNA methyltransferase proteins. (**A**) Phylogenetic analysis of DNA methyltransferase. The phylogenetic tree resolved four groups including the Dnmt1, Dnmt2, Dnmt3 and fungal-specific DMTs clan. Red, blue, dark green and green branches represent clans of the Dnmt1, Dnmt2, Dnmt3 and fungal-specific DMTs clan, respectively. (**B**) Structure of DNA methyltransferase proteins. The DNA methyltransferases were collected from 14 fungi, 5 mammals, 6 invertebrates, 2 chordatas and 1 plant. The conservative domain of C-5 cytosine methyltransferase was identified by InterProScan, and the domain architectures were visualized using software DOG 2.0. (**C**) Transcriptional level of *dmtA* in wild-type strain. The expression level of *DmtA* was compared to gene encoded histone H3-K79 methytransferease (AFL2G_08335). Gene expression levels at each time point were normalized (ΔΔCT analysis) to *β-tubulin*.

In total, 36 C-5 cytosine methyltransferase protein sequences from different species (14 fungi, 5 mammals, 6 invertebrates, 2 chordates and 1 plant) were downloaded from the National Center for Biotechnology Information (NCBI) protein search page using the key words “C-5 cytosine methyltransferase” (http://www.ncbi.nlm.nih.gov/protein/). These 36 conserved DNA methylase domains were analysed, and a neighbour-joining phylogenetic tree was constructed. The phylogenetic tree resolved four groups including Dnmt1, Dnmt2, Dnmt3 and a fungal-specific DMT clan, of which Dim-2 from filamentous fungi was classified in the same group with Dnmt1 from plants and animals (Fig. 1A). In vertebrates and invertebrates, Dnmt1, which plays a vital role in maintaining DNA methylation, has five conserved domains, namely a DMAP1-binding domain, a DNA (cytosine-5)-methyltransferase 1 replication foci, a Zinc finger (CXXC-type) domain, a Bromo adjacent homology domain and a C-5 cytosine methyltransferase domain (Fig. 1B). The other DNA methyltransferase, Dnmt3, usually has a PWWP domain, an ADD domain and a C-5 cytosine methyltransferase domain. The novel identified DNA methyltransferase Dnmt2, which only has a C-5 cytosine methyltransferase domain, functions mainly as a tRNA methyltransferase, but not as a DNA methyltransferase (Fig. 1A). The DNA methyltransferase DmtA identified in *Aspergillus* is highly divergent from the DNA methyltransferases that occur in species where DNA methylation has been validated. These proteins from *Aspergillus* also share a highly conserved structure (Fig. 1B, Figure S1). Of particular interest, we found that the DmtA proteins possessed by *Aspergillus* are closely related to two putative Masc1 DMTs in *A. immerses* and RID in *Neurospora*. Next, we investigated the transcriptional level of *dmtA* in the wild-type strain at different time points. Here, the gene AFL2G_08335 encoded histone H3-K79 methytransferease, which we are studying and showing important in *A. flavus* (data not shown), was used as a positive control for better understanding the expression pattern of *DMTA*. The results showed that *DmtA* transcripts were present at very low levels at the 24 h time point; however, during the time where AF production was high (after 48 h, Figure S2), the *dmtA* transcript level increased dramatically (Fig. 1C), indicating that DmtA might have some function(s) in *A. flavus*. Therefore, a gene knockout was carried out to study the function of DmtA in *A. flavus*.

### Generation of *A. flavus DmtA* knockout mutants and phenotypic characterization

**Figure 2:**
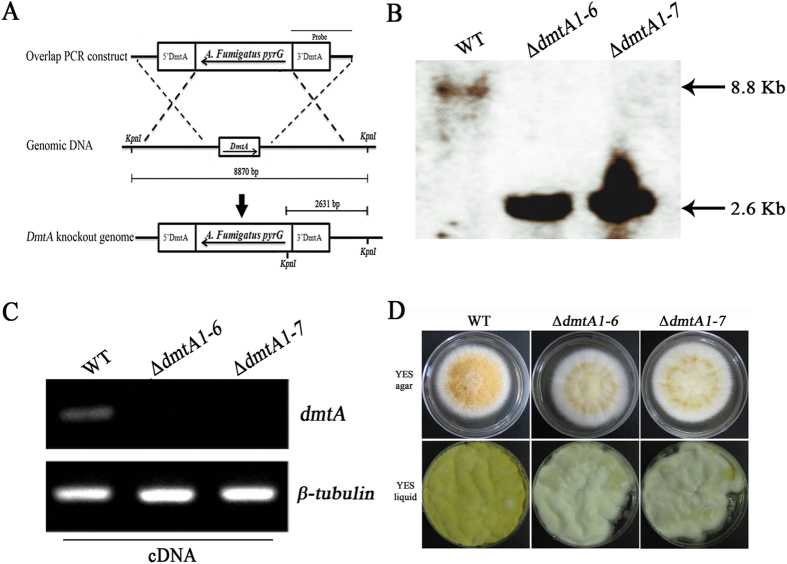
Construction of *dmtA* mutants and molecular analysis. (**A)** Schematic diagram of genomic region of the *dmtA* and *pyrG* genes. (**B)** Southern blot analyses of *dmtA* deletion mutants using PCR fragment of 3 flanking region as probe. Genomic DNAs were extracted from wild type and putative transformants. DNAs were digested with *Kpn* I and gel fractionated. The expected size is 2.6 kb for mutant and 8.8 kb for wild type (WT) strain. (**C)** RT**-**PCR verification of *dmtA deletion*. β-tubulin gene was used as a reference. (**D**) Morphological phenotypes of Δ*dmtA* mutants on YES agar media for 4 d and in YES liquid media for 5 d.

To gain insight into the potential function of *DmtA* in *A. flavus*, we first made full-length deletion mutants of the *DmtA* gene using *pyrG* selection. The schematic diagram of the genomic region of the *dmtA* and *pyrG* genes is shown in Fig. 2A. Among the many transformants obtained, most displayed identical phenotypes. Two uracil/uridine autotrophy isolates (Δ*dmtA1–6* and Δ*dmtA1–7*) were confirmed to be knockouts by Southern blot (Fig. 2B), and the failure of gene expression in Δ*dmtA* was also verified by RT-PCR (Fig. 2C). Δ*dmtA1–6* and Δ*dmtA1–7* were selected for further analysis. The colony phenotypes of the Δ*dmtA* mutants differed markedly from those of the control strain when grown on YES agar medium; they displayed a slightly flavescent conidial pigmentation compared with the yellow coloration of the wild-type line grown on the same medium, while the Δ*dmtA* mutants grown in YES liquid medium produced white fluffy mycelium (Fig. 2D) which is consistent with the phenotype produced by DNA methyltransferase inhibitor 5-azacytidine (5-AC) treatment^15^. However, the *dmtA* mutants started to produce some conidia after 7 days (data not shown). The difference between mutants and WT might be due to a delay of conidial formation.

### DmtA is important for conidiation in *A. flavus*

**Figure 3:**
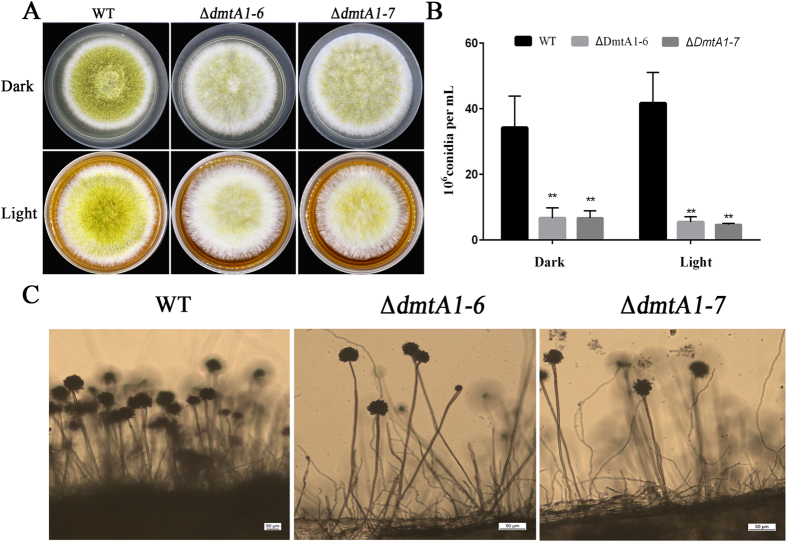
Deletion of dmtA altered colony morphology and conidiation. (**A)** Phenotypic characterization among the WT strain and the *dmtA* mutants in both dark and light incubation. (**B)** Deletion of *dmtA* resulted in conidiation reduction. The wild-type strain and Δ*dmtA* mutants were grown on YES agar for 3 d at 37 °C. (**C)** Conidia formation was observed under a light microscope at 12 h after induction with illumination. The asterisks **represents a significant difference level of P < 0.01.

To examine the role play of DmtA in fungal development and conidiation, 10^3^ conidia were pointed onto YES agar at 37 °C and grown for 3 days under both dark and light conditions. The results showed that when grown under such conditions, a significant decrease in conidiation production occurred in the Δ*dmtA* mutant colonies when compared with the wild-type line (Fig. 3A,B). For further analysis of the conidia defect, we examined the conidiophore formation, which showed that the Δ*dmtA* mutants produced fewer conidiophores than the wild-type line (Fig. 3C). The alterations observed in the conidiophore numbers for the Δ*dmtA* strain possibly resulted in decreased conidiation in the mutants. These results indicate that DmtA plays a role in conidiation in *A. flavus*.

### DmtA contributes to aflatoxin biosynthesis

**Figure 4:**
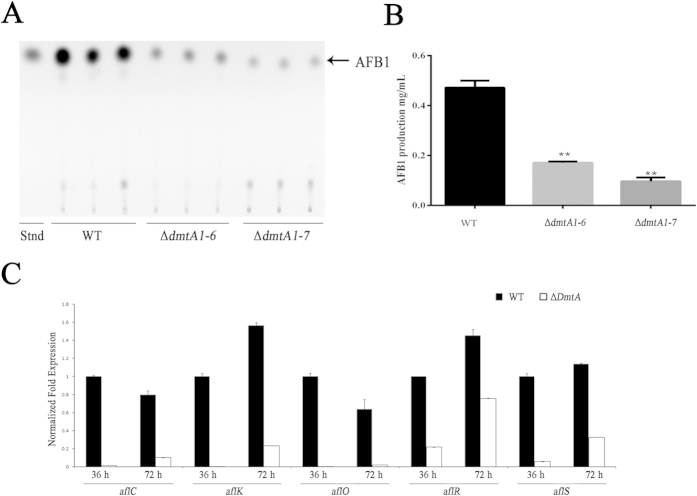
AF production and expression of AF biosynthetic genes in ΔdmtA mutants. (**A)** AF production of wild-type strain and Δ*dmtA* mutants was detected by TLC after cultured in YES liquid media for 5 d at 28 °C in the dark. (**B)** Quantification of AFB1 by High Performance Liquid Chromatography analisis. **(C)** Q-PCR result of AF biosynthetic genes (*aflC*, *aflK*, *aflO*, *aflR* and *aflS*) at 36 and 72 h. Gene expression levels at each time point were normalized (ΔΔCT analysis) to *β-tubulin*. The asterisks **represents a significant difference level of P < 0.01.

*A. flavus* is well-known as a saprophytic soil fungus because of its secondary AF metabolites, which are the most toxic and carcinogenic natural contaminants. To examine the effect of DmtA on AF biosynthesis, we tested AF production in the wild-type line and Δ*dmtA* mutants by thin layer chromatography (TLC) at 5 d, which showed a statistically significant decrease in the Δ*dmtA* mutants (Fig. 4A). For quantitative analysis of AF production, high-performance liquid chromatography (HPLC) was also used to confirm the presence of AF in the samples. The HPLC peak that eluted after 11.83 min corresponded to aflatoxin B1 (AFB1) and showed that AFB1 production in the wild-type strain was about 20-fold higher than that of Δ*dmtA* (Fig. 4B). These results indicate that inactivation of *DmtA* affected AF biosynthesis in *A. flavus*. To determine whether DmtA regulates AF biosynthesis at the transcript level, biosynthetic AF gene expression was analysed by qPCR at 36 and 72 h time intervals (Fig. 4C). We analysed three AF structure genes, *aflC* (*pksA*), *aflM* (*ver-1*) and *aflO* (*omtB*), and two globally regulated genes, *aflR* and *aflS*. Our current results showed that *aflC*, *aflK*, aflO, *aflS* and *aflR* were severely suppressed in the Δ*dmtA* mutants at the 36 h and 72 h time intervals (Fig. 4C). The transcript levels of these genes at the 72 h time interval increased concurrently, indicating that expression of these genes might be delayed and co-regulated in the ΔdmtA mutants. Interestingly, the transcript levels of *aflO*, which encodes O-methyltransferase B, were very low at the 36 h and 72 h time intervals. These results indicate that DmtA might regulate AF biosynthesis by repressing gene expression from the AF cluster.

### DmtA is unlikely to be involved in the hyper osmotic stress response

**Figure 5:**
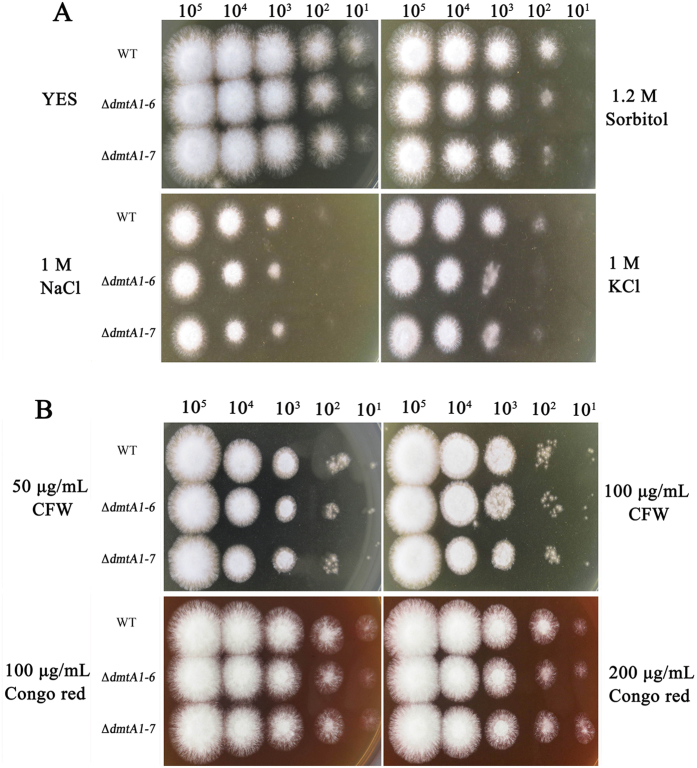
The effect of hyper osmotic stress and cell wall stress on Δ*dmtA* mutants. **(A)** Morphological phenotypes of WT and Δ*dmtA* mutants on YES agar in the presence of 1.2 mol/L sorbitol, 1M NaCl or 1M KCl in YES agar media. **(B)** Morphological phenotypes of wild type WT and Δ*dmtA* mutants on YES agar supplemented with 50 μg/ml Calcoflour white (CFW), 100 μg/ml CFW, 100 g/ml Congo Red or 200 g/ml Congo Red.

DNA methylation has been shown to play a vital role in many biological processes. Here, we explored the function of DmtA in the morphological development and AF production of *A. flavus*. To examine the potential roles for DmtA in environmental stress responses, we tested fungal sensitivity to hyperosmotic stress factors and cell-wall damaging agents. Growth of the Δ*dmtA* mutants in the presence of the osmotic stress agents NaCl and KCl was analysed. Interestingly, the mutant and wild-type lines showed almost the same sensitivities to hyper osmotic stress (1 mol/L NaCl, 1 mol/L KCl and 1.2 mol/L sorbitol) (Fig. 5A). Furthermore, the growth of the Δ*dmtA* mutant has been analysed in the presence of the cell-wall perturbing agents, Congo red and Calcofluor white. However, the Δ*dmtA* mutant was not sensitive to these cell wall damaging agents (Fig. 5B). According to our current study DmtA is probably not involved in resistance to hyper osmotic stress in *A. flavus*.

### DmtA may play a negative role in sclerotial production in *A. flavus*

**Figure 6:**
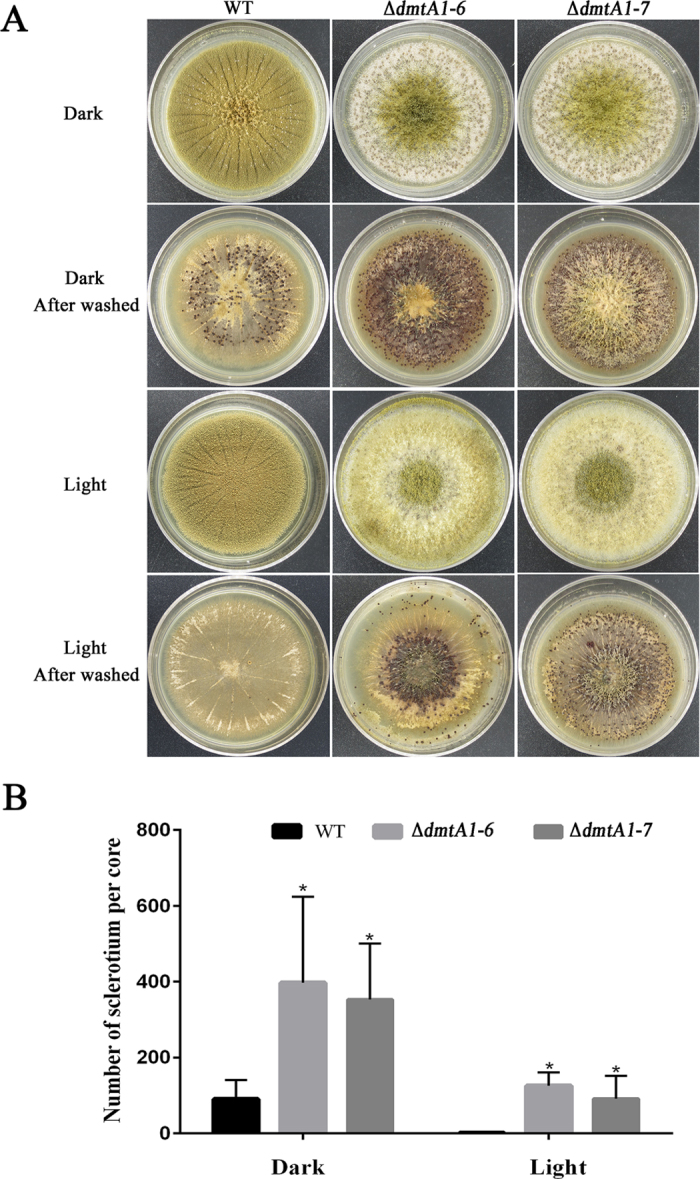
Effect of sclerotia formation among the WT and Δ*dmtA* mutants in both dark and light condition. **(A)** Deletion of *dmtA* increased sclerotial production. The wild-type strain and Δ*dmtA* mutants were grown on WKM agar for 7 days in both dark and light condition. The plates were sprayed with 75% ethanol to allow visualization of sclerotia. **(B)** Sclerotial production was counted from three replicates of WKM plates in (**A**). The asterisks *means significantly different (P < 0.05).

Studies have shown that sclerotial production is linked to AF synthesis^20^,^21^. To determine if loss of *DmtA* resulted in aberrations in sclerotial production, the strains were grown on sclerotial inducing Wickerham’s (WKM) agar under conditions of dark or light at 37 °C for seven days. After spraying with 75% ethanol to wash away the conidia, the sclerotial phenotype was visualized. To our surprise, unlike the negative effects on AFs production, our results showed that the Δ*dmtA* had almost quadruple the sclertotia production of the wild type (Fig. 6A,B). Additionally, fewer sclerotia were produced by Δ*dmtA* under conditions of light than when grown in the dark. These results indicate that DmtA possibly plays a negative role in the formation of sclertotia in *A. flavus*.

### Seed infection is altered in the DmtA mutants

**Figure 7:**
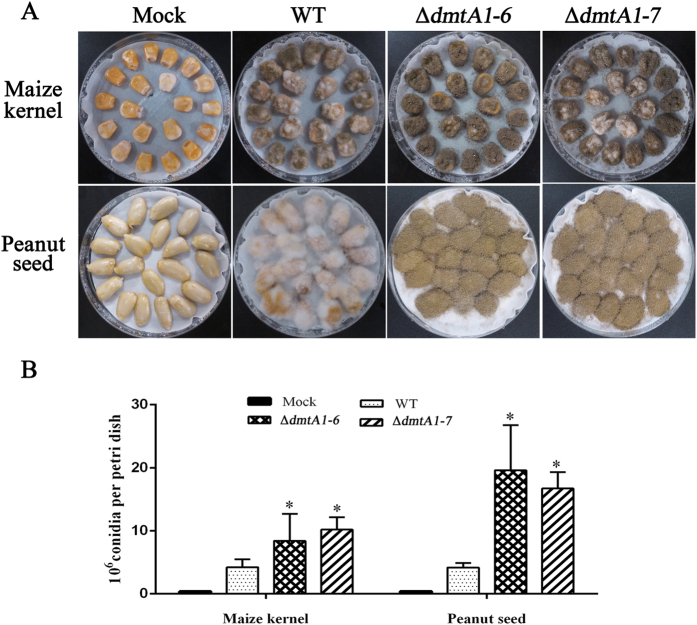
Host colonization of Δ*dmtA* mutants. (**A**) The WT strain and Δ*dmtA* mutants were grown on maize kernel and peanut seed for 5 d at 28 °C. (**B)** Conidia production was assessed from maize kernel and peanut cotyledons infected in (**A**). Values are the mean of three replicates and error bars represent standard errors. The asterisks *means significantly different (P < 0.05).

Although AF is not considered to be a virulence factor and changes in AF production have not been shown to be important in seed infections, we considered it possible that deletion of DmtA might affect seed infections. In this study, we examined the ability of the ∆*dmtA* strains to colonize peanut seeds and maize kernel. The wild-type strain tended to produce fluffy mycelium when colonizing peanut seeds and maize kernel (Fig. 7A), but the ∆*dmtA* strains appeared to grow more vigorously than the wild-type strain on crop seed (Fig. 7A). Moreover, we measured conidial production in the two strains on seed. Of interest, the ∆*dmtA* mutants produced statistically more conidia than the wild-type strain (Fig. 7B).

### DmtA is located in the nucleus and cytoplasm

**Figure 8:**
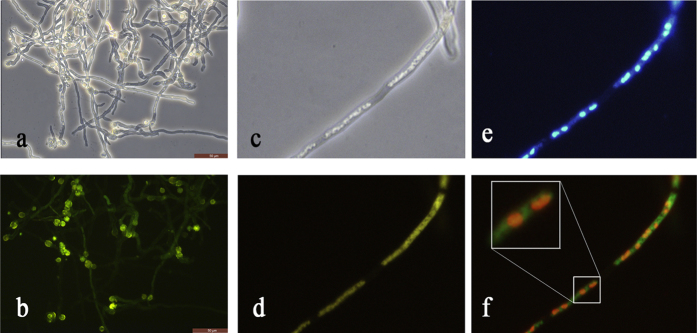
Localization pattern of DmtA-GFP. Subcellular location of DmtA. **(a,c)** the shapes of hyphae are shown by DIC imaging. **(b,d**) the location of DmtA-GFP. **(e)** Hyphae of wild-type strain was stained with 10 μg/mL 40′, 6-diamidino-2-phenylindole (DAPI) and examined by microscopy under UV light. **(f)** Merged photo of nucleus and DmtA-GFP.

So far, the subcellular location of DmtA has not been reported in *Aspergillus*. Therefore, to study the cellular location of DmtA, we generated a strain expressing a GFP tag at the C-terminus of DmtA (DmtA-GFP) under the control of its native promoter. During the time period of spore germination, DmtA::GFP accumulated in the tip of swellings (Fig. 8a,b). However, in the vegetative growth period, it showed a strong fluorescence signal in the nucleus and cytoplasm of the hyphae (Fig. 8d,f).

### DmtA might not function as a DNA methyltransferase in *A. flavus*

**Figure 9:**
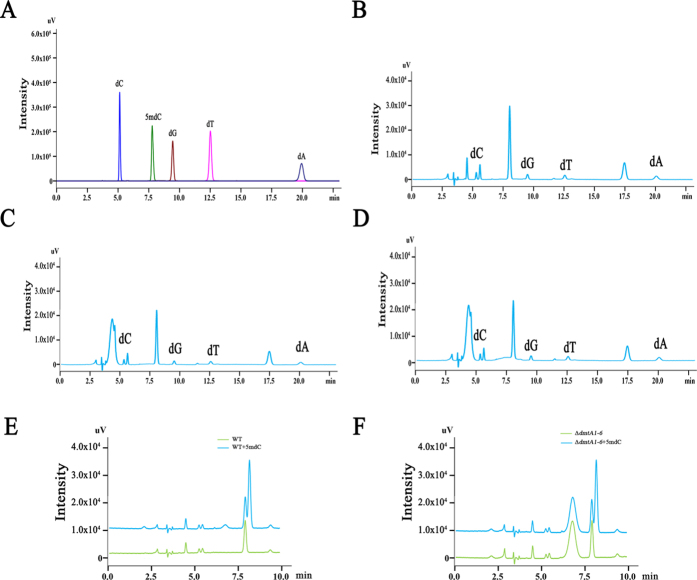
Detection of DNA methylation level in WT strain and Δ*dmtA* mutants genomic DNA. An eluent consisting of 10 mM KH_2_PO_4_ in 10% methanol adjusted to pH 4.7 with NaOH was used at a flow rate of 0.8 ml/min for separation of hydrolyzed DNA isolated from *A. flavus* wild-type (**B**) and Δ*dmtA* mutants (**C,D**). **(A)** Chromatogram of 5 standard deoxynucleotide. dA, dC, dG, 5mdC and dT represent 2′-deoxyadenosine, 2′-deoxycytidine, 2′-deoxyguanosine, 5-methyl-2′-deoxycytidine and 2′-deoxythymidine, respectively. **(E**) Chromatogram of hydrolyzed DNA isolated from *A. flavus* wild-type strain mixed with 10% 5mdC. **(F)** Chromatogram of hydrolyzed DNA isolated from Δ*dmtA1–6* mutant mixed with 10% 5mdC.

To determine whether changes in *A. flavus* development and AF biosynthesis induced by deletion of *DmtA* are caused by a reduction in DNA methylation, the 5-methyl-2-deoxycytidine (5-mdC) content of genomic DNA from *A*. *flavus* and the Δ*dmtA* mutants were both analysed by HPLC. The retention time for 2-deoxycytidine (dC) was 5.126 min and 7.771 min for 5-mdC (Fig. 9A). Here, we found that around the retention time of 7.97 min a peak was formed in both chromatograms from the wild-type and the Δ*dmtA* lines (Fig. 9B–D), and there was a stronger signal than dC. To validate if it was 5mdC, we mixed the hydrolysed DNAs isolated from the wild-type strain or Δ*dmtA* mutants with the 5mdC standard. However, two independent peaks were seen around the 7.7 min retention time (Fig. 9E,F), thus indicating that the substance with a retention time of 7.97 min in the chromatograms of the wild-type strain and Δ*dmtA* mutants was not 5mdC. These results suggest that there was no detectable 5-mdC in the genomic DNA of the *A. flavus* wild-type strain and the Δ*dmtA* mutants, which is consistent with the results of a former study^11^. Because of the low level of DNA methylation in the genomic DNA of *A. flavus*, it seems that DmtA might not function as a DNA methyltransferase in *A. flavus*.

## Discussion

DNA methylation, which is an important epigenetic modification, plays vital roles in regulating gene transcription, transposable element silencing, genome imprinting, X-chromosome inactivation and development in higher eukaryotes, and in genome defence in fungi^1^,^4^,^8^. In our previous study, we found that 5-azacytidine, which is a DNA methyltransferase inhibitor, caused a drop in AF production and concurrent morphological changes^15^, thereby hinting that DNA methylation or DMTs might perform roles in development and secondary metabolite biosynthesis in *A. flavus*, a fungus for which negligible DNA methylation has been found. In this study, to explore the potential role that DNA methylation might have, a C-5 cytosine methyltransferase DmtA from *A*. *flavus* has been idetifed from *A. flavus*. In addition to this, the knock-out mutants of *dmtA* we generated showed a decrease in AF production and conidiation, and a change in seed infection.

In filamentous fungi, the two putative fungal DMTs, Masc1 and RID, which have low DMTase activities, were shown to function in a genome defence system RIP in *Ascobolus immerses*, and MIP in *Neurospora*^8^,^11^,^12^. The putative DMT-like protein DMTA from *Aspergillus* shows high similarity with Masc1 and RID in the phylogenetic tree we generated. In *A. nidulans*, the Masc1/RID DMT-like protein identified was found to be essential for sexual development^8^. In our current study, we reported that instead of sexual development, a transient cellular process in *A. flavus*, inactivation of *dmtA* in *A. flavus* led to a dramatic decrease in conidiation production. An alteration in conidiophore numbers was also found in the Δ*dmtA* strain, which might result in decreased conidiation in the mutants.

In addition to conidiation, we found that *dmtA* affected the expression of genes involved in AF biosynthesis. As one of the most toxic and carcinogenic natural contaminants, AF is biosynthesized by an extremely sophisticated mechanism^19^. To the best of our knowledge, this is the first report on the effect of *dmtA* on secondary metabolism in fungi. Our results show that AF production was markedly blocked in the *dmtA* mutants, and the transcript levels of the structure genes *aflC*, *aflK* and *aflO*, together with the regulator genes, *aflR* and *aflS*, were severely suppressed compared with those of the wild-type strain. These data suggest that DmtA may be required for activating the AF gene cluster.

To address the effect of DmtA on *A. flavus* physiology and pathogenicity, we also examined sclerotia formation and host colonization in the Δ*dmtA* mutants. Interestingly, our results showed that sclerotial production in the Δ*dmtA* mutants dramatically increased compared with the wild-type line, a result differing from a previous study where enhanced sclerotial production was accompanied with increased AF production^22^. However, there is no direct evidence of a close link between sclerotial formation and AF biosynthesis. In this study, we found that deletion of the *dmtA* gene resulted in a drop in conidiation but enhanced sclerotial production, findings consistent with previous studies^22^,^23^. We also noted that Δ*dmtA* appeared more aggressive in tissue maceration compared with the wild-type line, which produced much more conidia on peanut and maize crops. A previous study showed that degradative enzymes such as lipase and esterase played a significant role in fungal infections of plants by *A. flavus*^23^. A change in the activities of these degradative enzymes might have occurred in the *DmtA* deletion mutants, leading to increased pathogenesis in hosts.

Nevertheless, it is interesting to consider why a gene known to be related to RIP in *Neurospora* would be quite so conserved and functional in *A. flavus*, which apparently lacks active RIP and DNA methylation. Because a negligible level of DNA methylation in the genomic DNA of *A. flavus* was observed, it appears that DmtA might not function as a DNA methyltransferase in *A. flavus*. However, the inactivation of this DMTA did result in changes in *A. flavus* development and AF biosynthesis. Studies have shown that DMTs are associated with the initiation of chromatin remodelling and gene regulation^24^,^25^. The effect of Dnmt1 on gene expression may depend on histone deacetylase activity via its N-terminal non-catalytic domain binding to histone deacetylases^26^. Meanwhile, a positive correlation between transcriptional activation of AF cluster genes and histone H4 acetylation has been found in *A. flavus*^27^. It also has been shown that inactivation of the sterigmatocystin gene cluster in *A. nidulans* requires epigenetic control by H3K9 methylation and heterochromatin protein-binding to establish a repressive chromatin structure^28^. In *Aspergillus*, the known global regulator of many secondary metabolites, LaeA, which also functions as a methyltransferase, plays a role in chromatin remodelling at the site of secondary metabolite gene clusters^29^,^30^. Because DNA methylation in *A. flavus* is negligible, DmtA might function in chromatin remodelling in a way that is dependent on other epigenetic modifications, in a manner similar to Dnmt1 or LaeA. Further research is needed to show the role of DmtA in chromatin remodelling in *A. flavus*.

In conclusion, our results provide evidence that DmtA, a putative C-5 cytosine methyltransferase, is essential for conidiation and AF metabolism in *A. flavus*. Moreover, inactivation of *dmtA* induced a change in seed infection, which produced statistically more conidia in crop seeds than the wild-type strain. Furthermore, no detectable DNA methylation in the genomic DNA of the *A. flavus* wild-type strain and Δ*dmtA* mutants was found. Thus, we propose that DmtA might not function as a DNA methyltransferase in *A. flavus*. The Δ*dmtA* mutant lines may be useful tools for exploring pre- or post-harvest control of AF in *A. flauvs*. Our results also provide valuable information that could advance our understanding of the epigenetic modification of AF biosynthesis in *A. flavus* and give a clue for furthermore study in the future.

## Materials and Methods

### Strain and culture conditions

*Aspergillus flavus* PTS Δ*ku70*Δ*pyrG*, a uracil auxotrophic, purchased from the Fungal Genetics Stock Center, School of Biological Sciences, University of Missouri, Kansas City, USA, was used for gene disruption. *A. flavus* was cultured on YES (2% yeast extract, 150 g/L sucrose, 1 g/L MgSO_4·_7H_2_O and 20 g/L agar) or YGTUU agar (5 g/L % yeast extract, 20 g/L glucose, 1 mL of trace element solution per litre of medium, 1 mg/mL uracil, 1 mg/mL uridine and 15 g/L agar) at 37 °C.

### DNA methyltransferase domain architecture and phylogenetic tree generation

DmtA, a C-5 cytosine methyltransferase in *A. flavus* was identified in the NCBI using the Basic Local Alignment Search Tool (http://blast.ncbi.nlm.nih.gov/Blast.cgi). Its coding sequence was cloned into the pET-28a prokaryotic expression vector (+) and sequenced. The sequence obtained was compared with the coding sequence reported in NCBI. Then, the conservative domain of the C-5 cytosine methyltransferase was identified using InterProScan^31^. To visualize the protein domain architectures, software DOG 2.0 was used^32^. The phylogenetic tree based on all the available DNA methyltransferase sequences from different organisms was constructed by MEGA5.0 software using the Neighbour-joining method. Bootstrap analysis was performed with 1000 replicates. The protein GI number of the DNA methyltransferase is shown as below: DMT(E. coli O26:H11), 608747058; DmtA(*A. oryzae*), 317148994; DmtA(*A. niger*), 145250405; DmtA(*A. nidulant*), 28208637; DmtA(*A. kawachii*), 358374015; DmtA(*A. fumigatus*), 846909269; DmtA(*N. fischeri*), 119467548; DmtA(*A. clavatus*), 119398323; DmtA(*A. flavus*), 220695028; Dim-2(*M. robertsii*), 629717833; Dim-2(*B. bassiana*), 667652773; Dim-2(*N. tetrasperma*), 350287792; Masc1(*A. immersus*), 2558956; RID(*N. crassa*), 20531189; RID(*N. tetrasperma*), 20531193; Dnmt1(*A. thaliana*), 15239810; Dnmt1(*D. rerio*), 190338613; Dnmt1(*H. sapiens*), 195927037; Dnmt1(*R. norvegicus*), 214010196; Dnmt1(*S. scrofa*), 73853882; Dnmt1(*M. musculus*), 148693193; Dnmt1(*O. aries*), 57164173; Dnmt1(*X. laevis*), 148225023; Dnmt1(*B. mori*), 112983430; Dnmt2(*A. thaliana*), 18420929; Dnmt2(*S. frugiperda*), 406868804; Dnmt2(*S. scrofa*), 242253856; Dnmt2(*D. elanogaster*), 116007318; Dnmt3(*D. rerio*), 190337984; Dnmt3(*A. mellifera*), 298677086; Dnmt3(*D. pulex*), 321467881; Dnmt3B(*D. rerio*), 70887603; Dnmt3A(*D. rerio*), 688595232; CMT(*A. thaliana*), 15222449; CMT(*M. domestica*), 658309668.

### Construction of Δ*dmtA* mutants

All the primers used in this study are listed in Table 1. To generate Δ*dmtA* mutants, a 1226 bp fragment upstream from *DMTA* was amplified with *dmtA*/P1 and *dmtA*/P3 primers. Next, a 1049 bp fragment downstream from *DMTA* was amplified with *dmtA*/P4 and *dmtA*/P6 primers. To generate the fragment containing the upstream fragment, the *pyrG* selectable marker and downstream fragment were added sequentially, using the PCR fusion approach and the PCR program described by Szewczyk^33^. To generate the *dmtA* mutants, the fusion PCR product was transformed into protoplasts of the wild-type CA14 (Δ*ku70*Δ*pyrG*).

### Preparation of protoplasts and fungal transformation

In the initial transformation, the culture of spores harvested from YGTUU plates was inoculated into 150 ml of YGTUU liquid medium^34^,^35^,^36^, and the resultant culture was shaken at 180 revolutions/min for about 12 h at 37 °C. The cultures were collected on sterile filter paper, transferred to a 250 mL flask, and resuspended in 20 mL of filter-sterilized enzyme solution (10 mM NaH_2_PO_4_, pH5.8, 20 mM CaCl_2_, with 0.2 ml β-glucuronidase at 8500 U/mL, 200 mg lysing enzyme from *Trichoderma harzianum* (Sigma, MO, USA), 50 mg Driselase (Sigma) and 1.2 M NaCl). Digestion was performed at 70 revolutions/min at 30 °C for 3.5–4 h. Protoplasts, harvested by filtration through miracloth, were collected using a micro centrifuge. The protoplasts obtained were washed twice with STC solution (1.2 M sorbitol, 50 mM CaCl_2_, 50 mM Tris-HCl, pH7.5, autoclaved and stored at 4 °C).

Fungal transformation was performed as previously described with minor modifications^33^,^34^. Polyethylene glycol (PEG) buffer (50% PEG4000, 0.6 mM KCl, 10 mM NaH_2_PO_4_, pH5.8, 50 mM CaCl_2_ and 10 mM Tris-HCl, pH7.5) was substituted. The transformation mixture was mixed well with 20 mL of regeneration medium (Czapek’s Agar: 50.0 g/L of Czapek Solution Agar (BD Bioscience, NJ, USA), 1.0 M sucrose and 0.5% agar), plated onto selective plates, and 10 ml of regeneration medium (1.5% agar) was poured in the first layer. The plates were incubated at 37 °C for 2–3 d. Uracil/uridine autotrophy transformants were screened by PCR with primers *dmtA*/UA and P801/R and primers *dmtA*/OF and *dmtA*/OR (Table 1. PCR-positive isolates were verified by RT-PCR. Subsequently, knockout candidates were further verified by Southern blot with the Digoxigenin High Prime DNA Labeling and Detection Starter Kit I (Roche, Basel, Switzerland).

### Physiology experiments

Conidial production, sclerotial formation and relative colony diameter measurements were recorded for the wild-type and Δ*dmtA* strains. In order to eliminate the effect of illumination on *A. flavus* development, the physiology experiments were performed under dark and light condition. The diameter was measured from point inoculation of a 1 μL of a 106 spores/mL suspension of *A. flavus* conidia onto YES media. To analyse conidia production, YES media was overlaid with 5 mL of a 106 spore/mL suspension of *A. flavus* conidia in molten agar according to the former description by Shubha^23^. Cultures were grown for 5 d at 37 °C, and three 1.5 cm diameter cores were harvested from the centre of each plate and homogenized in 3 mL of distilled water. The spore number was counted haemocytometrically. For sclerotial production analysis, we used sclerotial inducing WKM medium^37^. Cultures were grown at 37 °C for 5 d under dark and light conditions. The plates were then sprayed with 75% ethanol to kill and wash away conidia to aid in enumeration of sclerotial.

### Seed infections

The ability of the Δ*dmtA* mutants to infect crop seeds was assayed as described previously^23^,^38^. Then, peanut cotyledons were inoculated with a 105 spores/mL of *A. flavus* conidia for 30 min, with continuous shaking (50 revolutions/min). A blank control was performed by immersing the cotyledons in sterile water. Twenty treatments were placed in petri dishes lined with two pieces of moist filter paper to maintain high humidity. Each treatment was performed with five replicates. Cotyledons were incubated at 28 °C for 5 d under dark conditions. The filter paper was moistened daily. Maize seeds were inoculated similarly to the peanut seeds. After incubation for 5 d, the peanut and maize seeds were harvested in 50 mL Falcon tubes, weighed, and then vortexed for 2 min to release the spores in 15 mL of sterile water supplemented with 0.05% Tween 80. Conidiation was counted haemocytometrically.

### AF analysis

To analyse AF production, a 5 mL aliquot of a 10^6^ spore/mL suspension of *A. flavus* conidia was incubated in YES medium in the dark at 28 °C for 5 d. AF was extracted from the 500 μL filtrate with an equal volume of chloroform. The chloroform layer transferred to a new 1.5 mL tube was evaporated to dryness at 70 °C. Next, TLC was used to analyse AF biosynthesis. A solvent system consisting of acetone and chloroform (1: 9, v/v) was used, and the TLC result was observed under ultra violet (UV) light at 365 nm.

For quantitative analysis of AF production, HPLC (High Performance Liquid Chromatography) was also used to confirm the presence of AF in the samples. The AF extract was filtered (0.22 mm) and analysed by HPLC (Agilent Series 20AD XR, Agilent Technologies, Santa Clara, CA, USA) on a MYCOTOX™ column (Catalog NO. 1612124, 5 m column 25 × 4.6 mm) at 42 °C. The column was equilibrated in the running solvent (56: 22: 22 water-methanol-acetonitrile), and 20 μL was injected and run isocratically for 14 min with 100% running solvent at a flow rate of 1.0 mL/min. AF was detected by a fluorescent detector with an excitation wavelength of 365 nm and emission wavelength of 455 nm.

### Construction of the DmtA-GFP vector

To localize DmtA, the 2644-bp fragment including the 1204-bp predicted promoter region and 1440-bp *dmtA* coding sequence was amplified using *dmtA*-GFP/F and dmtA-GFP/R primers (Table 1). The resultant 2644-bp PCR product was cloned into the pKNTG vector, which has a GFP tag and pyrG selectable marker, using a pEASY-Uni Seamless Cloning and Assembly Kit (TransGen Biotech, Beijing, China) to generate the pKN-DmtA-GFP vector. The pKN-*dmtA*-GFP vector was transformed into the CA14 Δ*ku70*Δ*pyrG* strain, and the transformants were PCR-verified.

### Reverse transcriptase (RT)-PCR and quantitative real-time PCR

Both wild-type and Δ*dmtA* mutant mycelia were harvested at growth stages (36 h and 72 h incubated on YES Agar). RNA molecules were isolated with TRIzol reagent (Biomarker Technologies, Beijing, China) and purified with the DNA-free kit (TransGen Biotech, Beijing, China). TransScript® All-in-One First-Strand cDNA Synthesis SuperMix was used to synthesize the first strand cDNA, and qRT-PCR was performed with the Thermo Fisher Scientific Real-time PCR System (Finland) using TransStart Top Green qPCR SuperMix (TransGen Biotech, Beijing, China). In the quantitative real-time PCR, AF *aflC*, *aflK* and *aflO* structural genes and *aflR* and *aflS* regulator genes were amplified by the primer pairs shown in Table 2. As an endogenous control, the β-tubulin gene was amplified with 9F/9R primers. The relative quantification of the transcripts was calculated by the 2^−ΔΔCt^ method^39^. All qRT-PCR assays were conducted with technical triplicates for each sample, and the experiment was repeated twice.

### DNA methylation detection

DNA isolation and digestion were performed as described previously^15^. After complete digestion, the digested DNAs were analysed by HPLC. An Agilent Cl8 Zorbax XDB column (150 mm × 4.6 mm, 5 mm, Agilent) was used to separate the digested DNAs. Elution was performed at 0.8 mL/min with a mobile phase of 10 mmol per L KH_2_PO_4_ and 10% methanol (v/v) (pH 4.7). The 10 μL DNA hydrolysate sample injected into the HPLC system was detected at 280 nm with a UV detector.

## Additional Information

**How to cite this article**: Yang, K. *et al.* The DmtA methyltransferase contributes to *Aspergillus flavus* conidiation, sclerotial production, aflatoxin biosynthesis and virulence. *Sci. Rep.* **6**, 23259; doi: 10.1038/srep23259 (2016).

## Supplementary Material

Supplementary Information

## Figures and Tables

**Table 1:** Gene-specific primers used for gene knock-out.

Primers	Sequence(5′-3′)	Application
*dmtA*/P1	CCAGCAGCATCTTGACCTC	*dmtA* deletion and probe
*dmtA*/P3	GGGTGAAGAGCATTGTTTGAGGCAACACGATGGTAGGGAACTG	
*dmtA*/P4	GCATCAGTGCCTCCTCTCAGACCCTCTTTCGGACCTTGCG	
*dmtA*/P6	CAGCAGTCATTGTAATCCAGCC	
*dmtA*/P2	TTCAAGGCTTCCAATCCACAG	
*dmtA*/P5	AGAGGCAAGGGAGAAGAAGC	
PyrG/F	GCCTCAAACAATGCTCTTCACCC	*dmtA* deletion
PyrG/R	GTCTGAGAGGAGGCACTGATGC	
*dmtA*/UA	AGCCTCGGCATTCGCACTG	*dmtA* mutant screen
P801/R	CAGGAGTTCTCGGGTTGTCG	
*dmtA*/OF	TAGTGAATCCCAACCACCTTT	*dmtA* mutant screen
*dmtA*/OR	CGTTCGCACCCTCTATTGTAT	
*dmtA*-GFP/F	CCTCGAGGTCGACGGTATCGATGCATCGAATATGAGATCAGGTC	GFP fusion
*dmtA*-GFP/R	CTGCAGGCATGCAAGCTTATCGATCCCAGAAGCAATGACGAGG	

**Table 2:** Gene-specific primers used for RT-PCR.

Primers	Sequence(5′-3′)	Application
*aflC*/QF	GTGGTGGTTGCCAATGCG	qRT-PCR
*aflC*/QR	CTGAAACAGTAGGACGGGAGC	
*aflK*/QF	GAGCGACAGGAGTAACCGTAAG	qRT-PCR
*aflK*/QR	CCGATTCCAGACACCATTAGCA	
*aflO*/QF	GATTGGGATGTGGTCATGCGATT	qRT-PCR
*aflO*/QR	GCCTGGGTCCGAAGAATGC	
*aflS*/QF	CGAGTCGCTCAGGCGCTCAA	qRT-PCR
*aflS*/QR	GCTCAGACTGACCGCCGCTC	
*aflR*/QF	AAAGCACCCTGTCTTCCCTAAC	qRT-PCR
*aflR*/QR	GAAGAGGTGGGTCAGTGTTTGTAG	
*dmtA*/QF	AAGAGGCGGCGACAGTATA	qRT-PCR
*dmtA*/QR	GATAGGTAGAGGTTGCGTGTTC	
β-tubulin/QF	TTGAGCCCTACAACGCCACT	qRT-PCR
β-tubulin/QR	TGGTTCAGGTCACCGTAAGAGG	
