# Supplementary material for: The DmtA methyltransferase contributes to Aspergillus flavus conidiation, sclerotial production, aflatoxin biosynthesis and virulence
Source: Sci Rep. 2016 Mar 16;6:23259. doi: 10.1038/srep23259 (PMC4793245; doi:10.1038/srep23259)
Supplement: Supplementary Information [file srep23259-s1.doc]

**The DmtA methyltransferase contributes to *Aspergillus flavus* conidiation, sclerotial production, aflatoxin biosynthesis and virulence**

Kunlong Yang^§^, Linlin Liang^§^, Fanlei Ran, Yinghang Liu, Zhenguo Li, Huahui Lan, Peili Gao, Zhenhong Zhuang, Feng Zhang, Xinyi Nie, Shimuye Kalayu Yirga,Shihua Wang*

Key Laboratory of Pathogenic Fungi and Mycotoxins of Fujian Province, Key Laboratory of Biopesticide and Chemical Biology of Education Ministry, and School of Life Sciences, Fujian Agriculture and Forestry University, Fuzhou 350002, China

§These authors contributed to this work equally.

***** Author correspondence to Shihua Wang. E-mail: [wshyyl@sina.com](mailto:wshyyl@sina.com); Tel and Fax: +0086 (591) 87984471


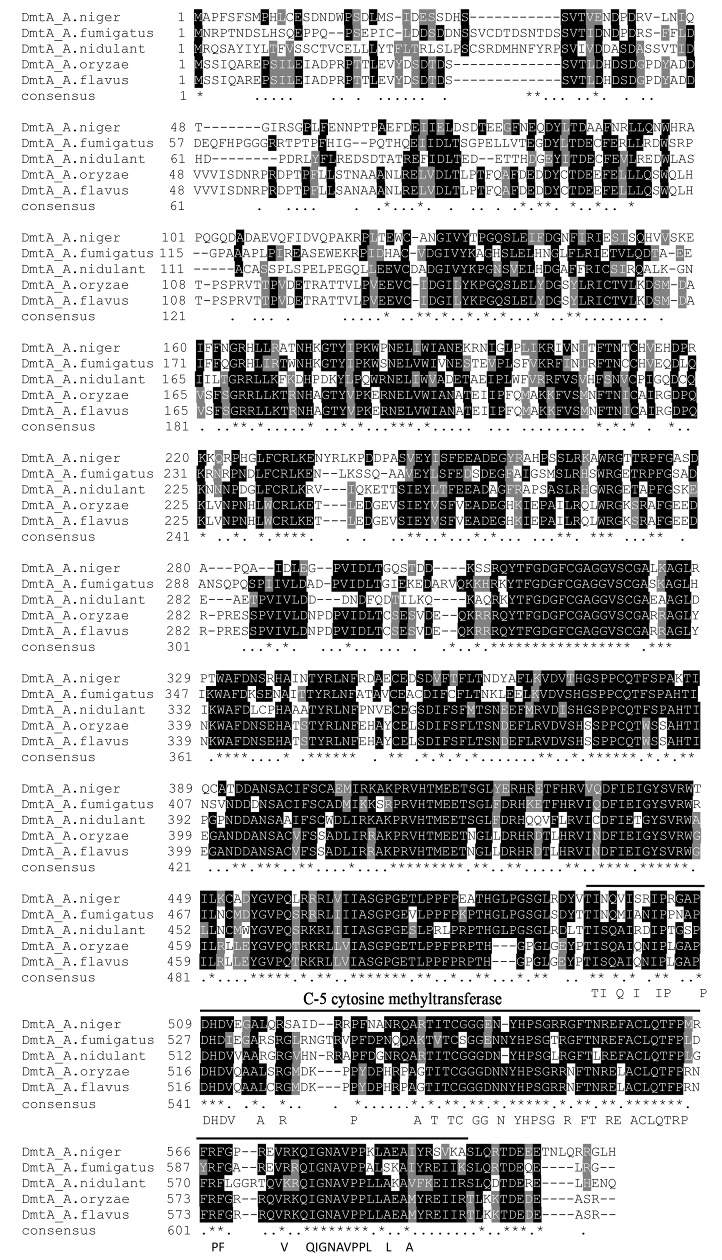


**Figure S1.** **Sequence alignment of *Aspergillus* DmtA proteins.** Clustal W and Boxshade (http://bioweb.pasteur.fr/seqanal/interfaces/boxshade.html) were used to analyze the predicted protein sequences. The conserved amino acid residues are shown in shaded black, whereas similar residues are shown in gray. Consensus amino acids are marked with asterisk (*).


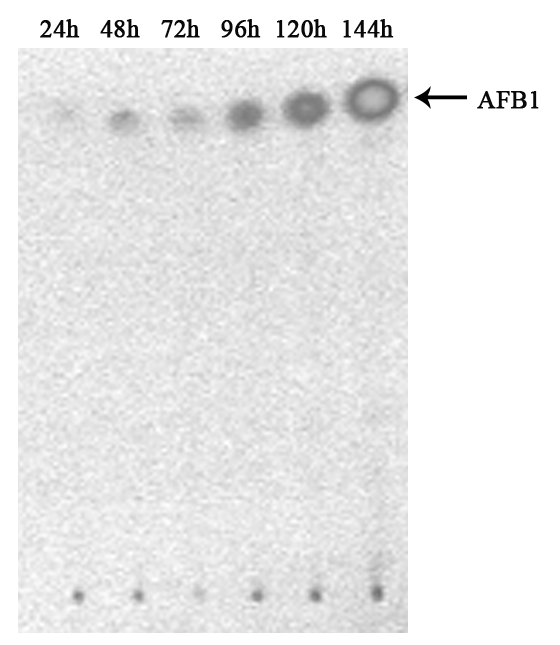


**Figure S2. Aflatoxin production at different time points.** AF production of wild-type strain was detected by TLC after cultured in YES liquid media for 24 h to 144 h at 28℃ in the dark.
